# Successful Integration of Recombinant Human Bone Morphogenetic Protein-2 (rhBMP-2)-Coated Dental Implant: A Case Report

**DOI:** 10.7759/cureus.79281

**Published:** 2025-02-19

**Authors:** Mayur Kaushik, Aprajita Srivastava, Shivi Khattri

**Affiliations:** 1 Department of Periodontology and Implantology, Subharti Dental College and Hospital, Swami Vivekanand Subharti University, Meerut, IND

**Keywords:** allograft, immediate implant placement, osseointegration, peri-implant bone regeneration, rh-bmp2

## Abstract

This case report describes the use of a recombinant human bone morphogenetic protein-2 (rhBMP-2)-coated dental implant for immediate placement in a 65-year-old male with a nonrestorable maxillary right first premolar. Due to the compromised condition of the tooth, extraction was necessary, and immediate implant placement was considered appropriate based on the patient’s dental and overall health. The rhBMP-2-coated implant was selected to enhance bone regeneration and promote osseointegration in the severely resorbed alveolar ridge. Over a six-month follow-up period, the patient exhibited successful implant integration, minimal postoperative complications, and favorable functional and aesthetic outcomes.

## Introduction

Dental implants have become the most reliable and standard treatment modality for replacing missing teeth due to their long-term predictability, functionality, and aesthetic outcomes [1]. However, one of the primary challenges in implantology is insufficient alveolar bone volume, particularly in patients with significant bone resorption resulting from tooth loss or periodontal disease. In such cases, traditional implant placement often requires bone augmentation procedures to ensure proper osseointegration [2].

Teeth requiring extraction due to severe decay, failed endodontic treatment, or crown fractures are commonly replaced with dental implants [3]. However, immediate implant placement presents several challenges, including achieving adequate primary stability, assessing bone quality and quantity in the extraction socket, ensuring proper implant positioning, and mitigating the risk of infection in the fresh extraction site [4]. When the extraction socket has reduced bone volume due to three-walled, two-walled, or one-walled defects, careful surgical management is essential. In such cases, simultaneous bone augmentation with immediate implant placement can be a viable approach. Bone augmentation can be performed using bone grafts, barrier membranes, growth factors, or their combinations [5].

Autografts have been successfully combined with xenografts for peri-implant bone regeneration (PIBR) [6]. Recombinant human bone morphogenetic proteins (rhBMPs) are powerful growth factors known to induce ectopic bone formation [7]. Specifically, rhBMP-2 has been widely studied for its ability to stimulate osteoblastic activity and facilitate bone defect healing [8]. The combination of xenografts with rhBMP-2 has also been explored for PIBR.

Given the potential benefits of rhBMP-2, it was coated onto the surface of a dental implant and combined with freeze-dried bone allograft (FDBA). A study by Lim et al. demonstrated favorable results using this combination for maxillary sinus floor elevation. However, there is limited literature on the use of FDBA with rhBMP-2 for PIBR [9].

Therefore, this case report aims to evaluate PIBR using a combination of FDBA and rhBMP-2 during immediate implant placement.

## Case presentation

**Figure 1:**
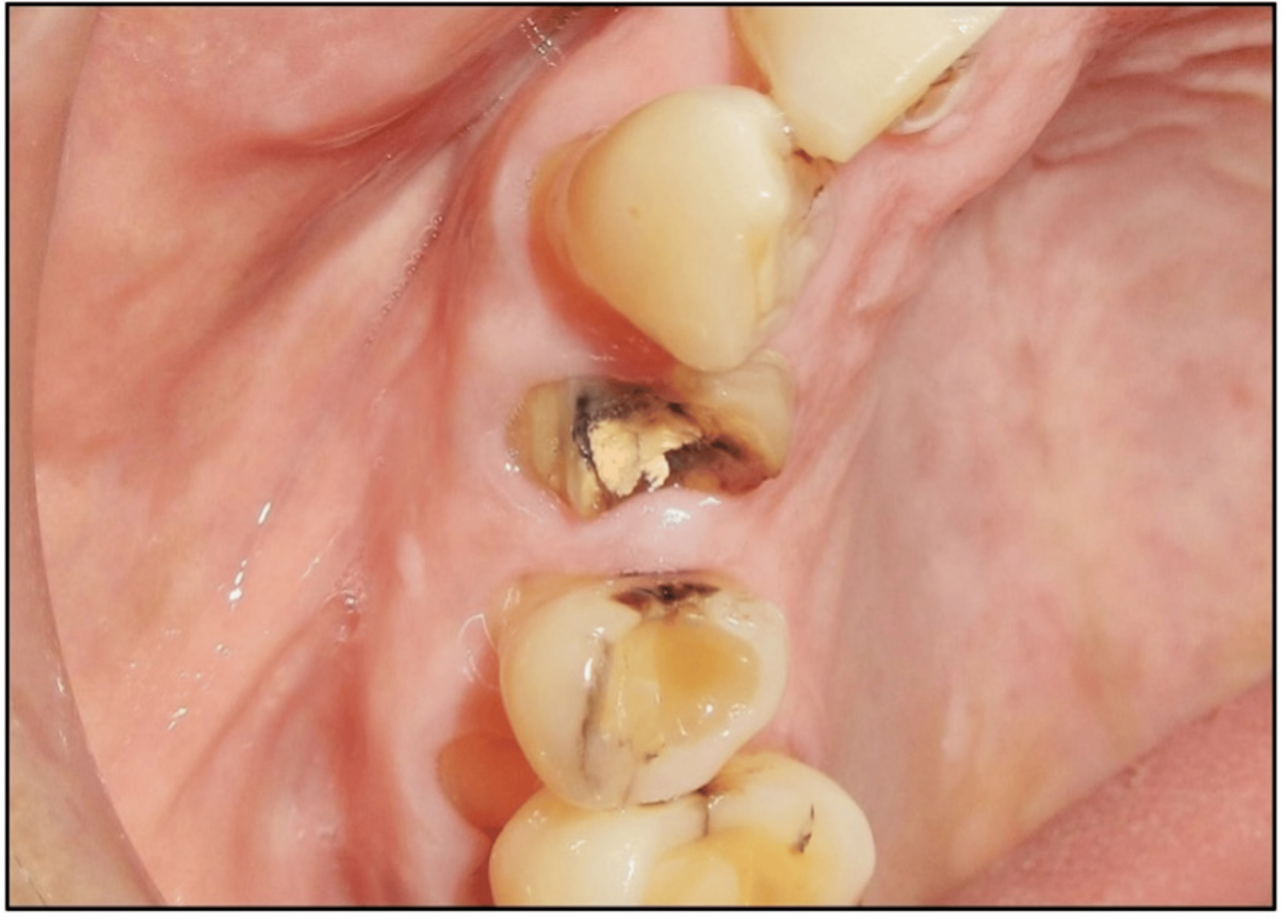
Preoperative image showing a fractured crown of a previously endodontically treated maxillary right first premolar (#14)

A 67-year-old male was referred to the OPD at Subharti Dental College and Hospital, Meerut, India, with a complaint of a fractured crown in the cervical region of tooth #14 (ADA numbering system) (Figure 1). The patient was a nonsmoker, systemically healthy, and not on any medications. He reported a history of endodontic treatment for the same tooth but had not undergone any previous periodontal surgery.

**Figure 2:**
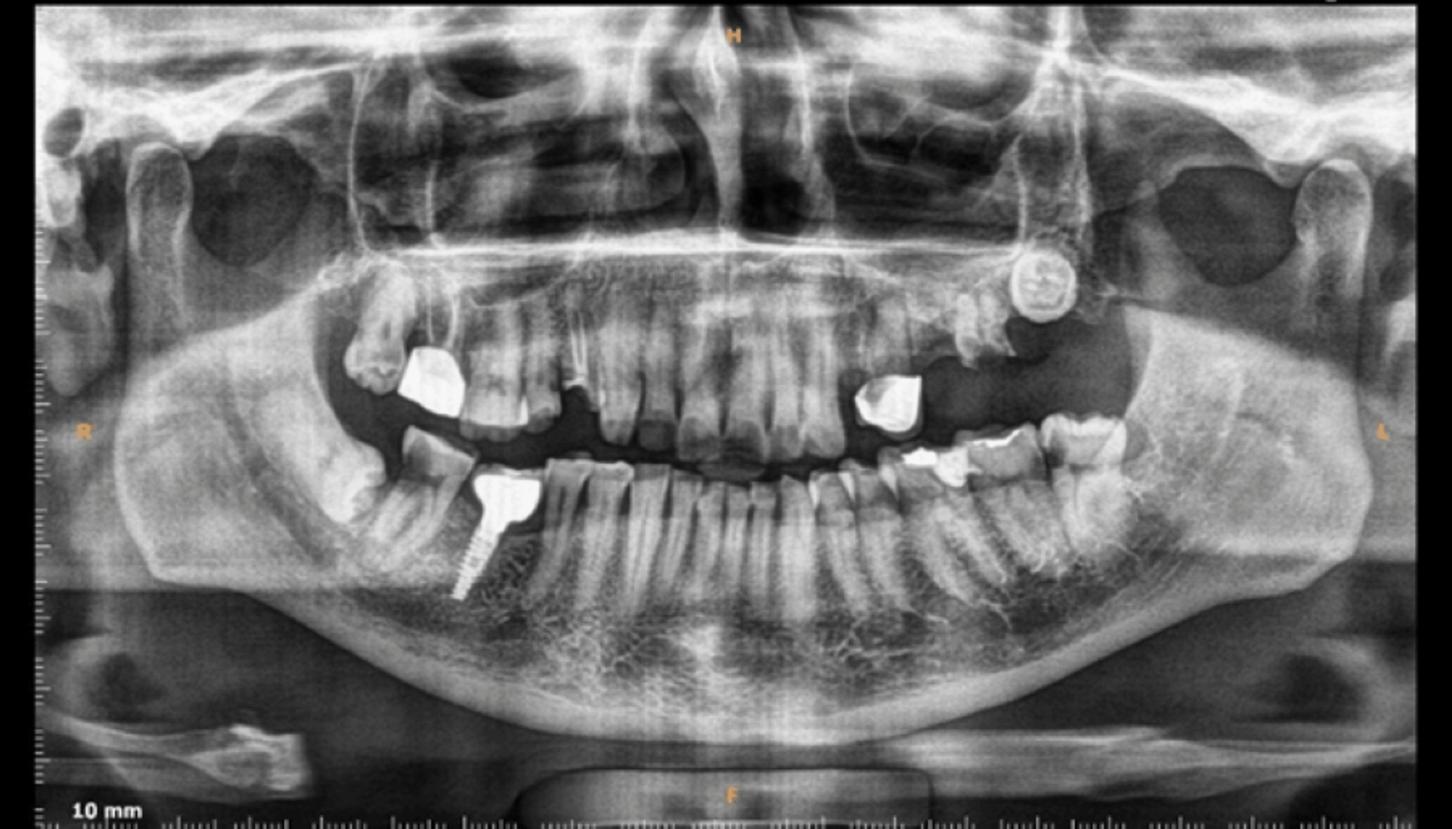
Preoperative orthopantomogram

**Figure 3:**
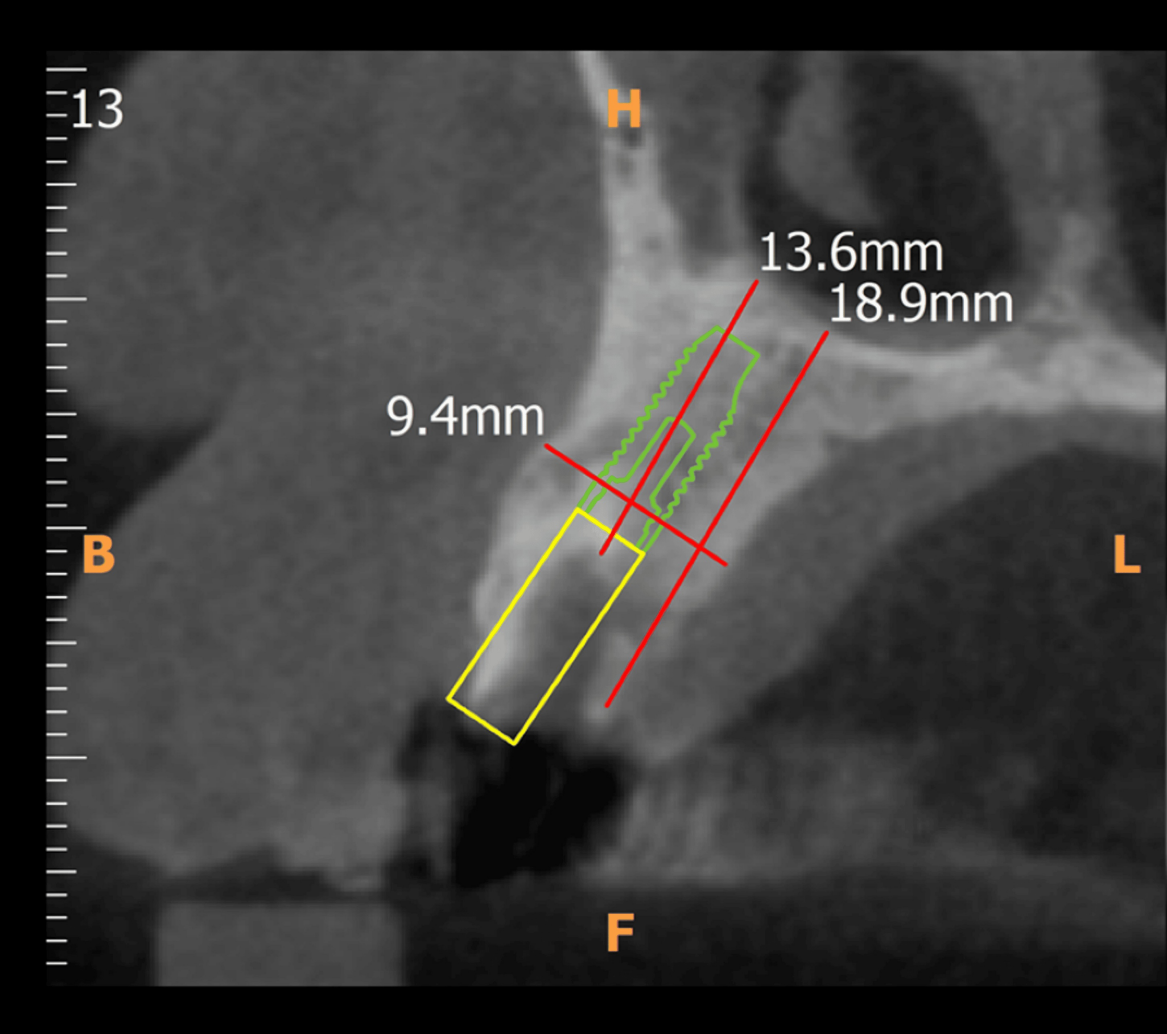
Preoperative CBCT CBCT, cone beam CT

The oral examination revealed that the patient was periodontally healthy. He was offered the option of re-treatment for the affected tooth; however, he opted for tooth replacement instead. Clinical and radiographic examinations confirmed adequate bone support with no signs of periapical or periodontal pathology (Figure 2). Consequently, cone beam CT (CBCT) was advised to assess bone availability and predetermine the angulation for implant placement. Figure 3 shows the preoperative CBCT of the implant site.

Thus, atraumatic extraction followed by immediate implant placement and PIBR was planned to promote bone regeneration and ensure the long-term stability of the implant.

Case management

Phase I periodontal therapy was completed before implant placement. Following this, informed consent was obtained from the patient.

Medication was prescribed one day prior to the procedure, including amoxicillin and deflazacort. Additionally, one hour before surgery, the patient was administered rabeprazole, diclofenac, and serratiopeptidase.

**Figure 4:**
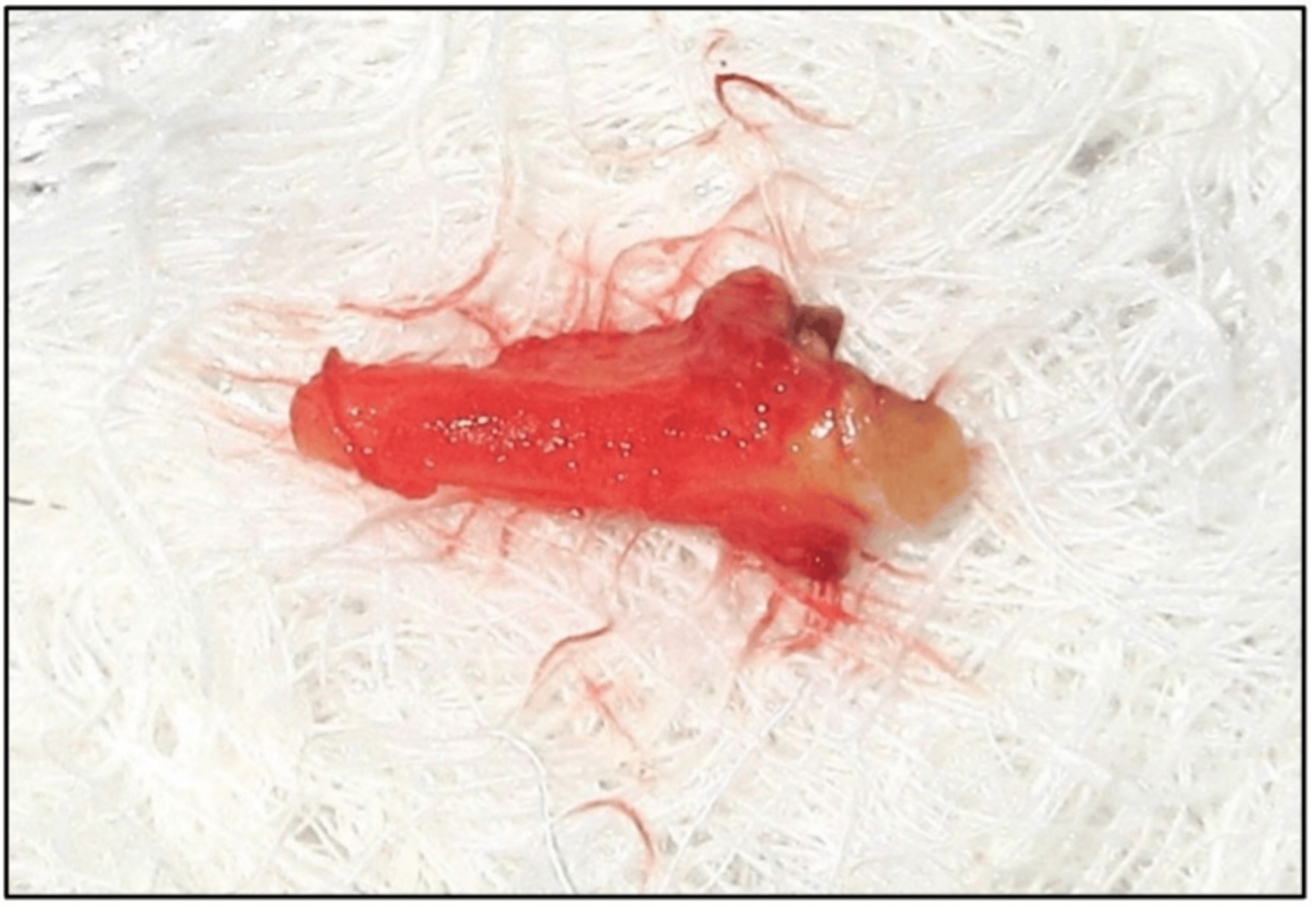
Extracted tooth

**Figure 5:**
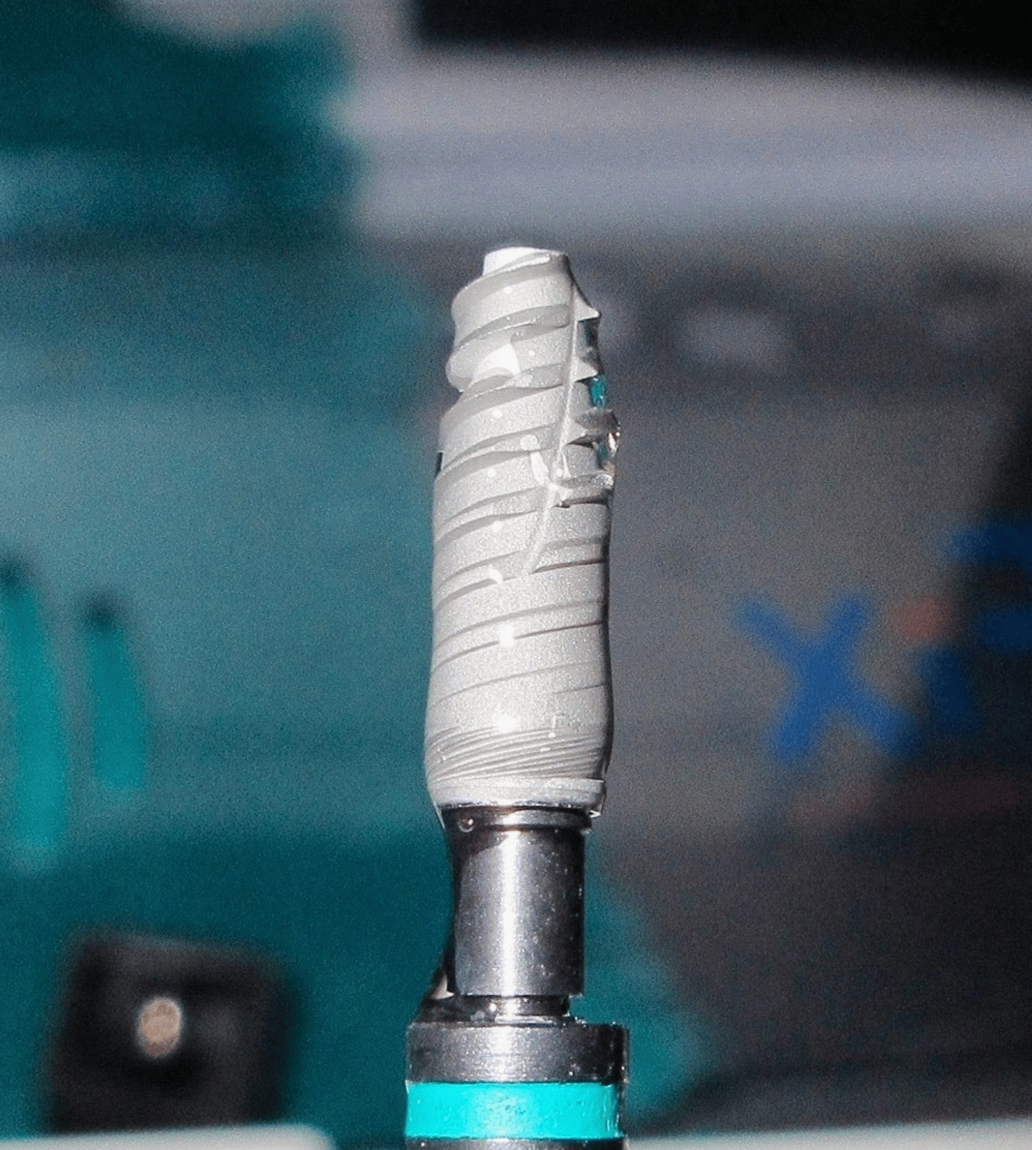
Implant surface coated with rhBMP-2 rhBMP-2, recombinant human bone morphogenetic protein-2

Local anesthesia (4% articaine with epinephrine 1:100,000; Septanest, Septodont, Saint-Maur-des-Fossés, France) was administered. Tooth #14 was then extracted atraumatically (Figure 4), followed by flap reflection. The osteotomy site was prepared, and a 3.5 × 11.5 mm implant (i-Fix Pro^®^, Kamal Medtech, Faridabad, India) was selected. Before implant placement, both the osteotomy site and implant surface were coated with rhBMP-2 (Figure 5) at a concentration of 0.5 μg/mL, stored at 4°C (PerioBiologics LLP, Hyderabad, India). The preparation of rhBMP-2 was performed as described by Chandra et al. [10].

**Figure 6:**
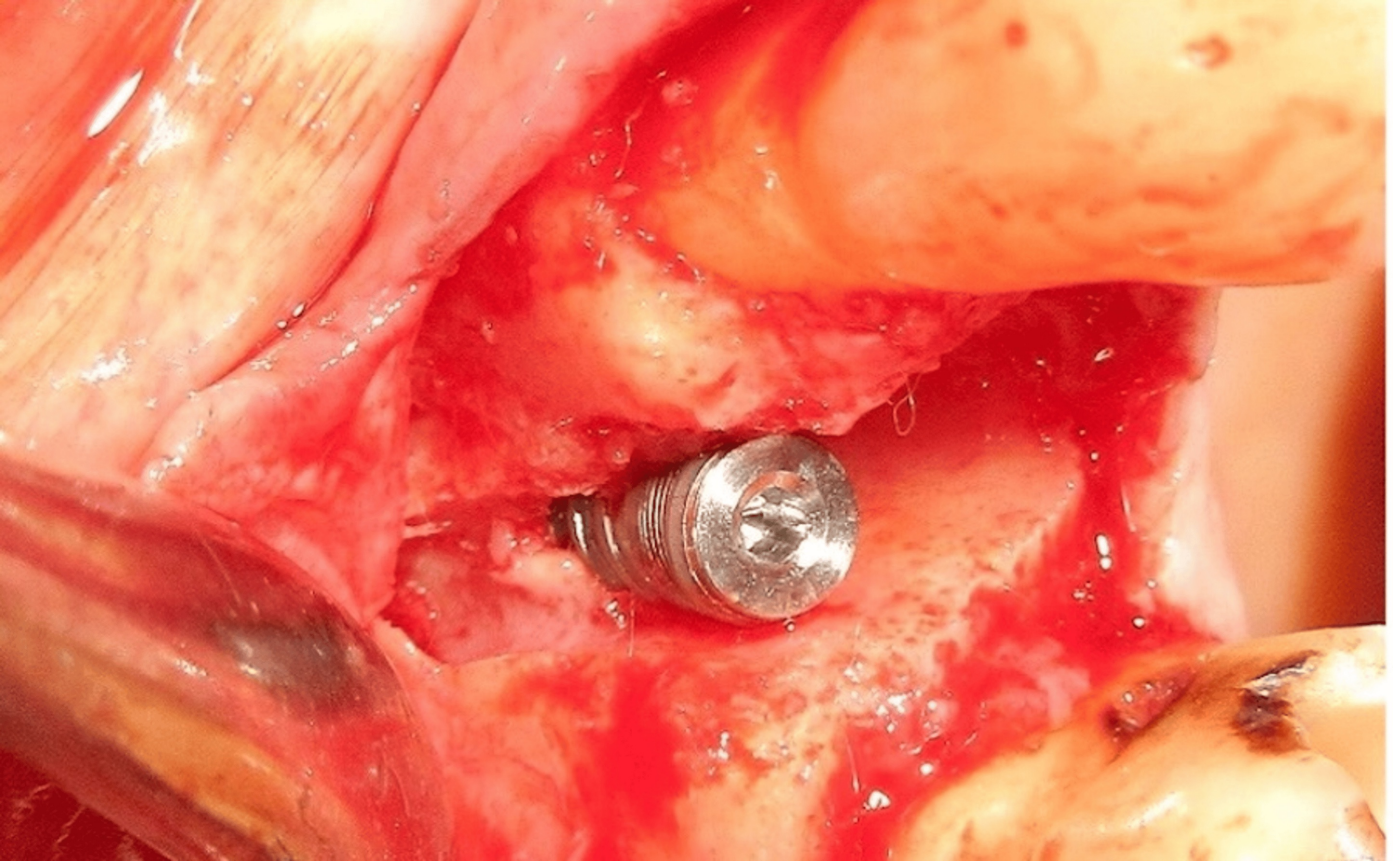
Implant placed at the osteotomy site of the maxillary right first premolar (#14)

**Figure 7:**
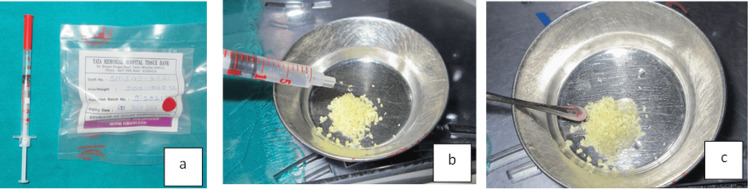
(a) FDBA and rhBMP-2; (b) Mixing of rhBMP-2 with FDBA; and (c) rhBMP-2-activated bone graft FDBA, freeze-dried bone allograft; rhBMP-2, recombinant human bone morphogenetic protein-2

**Figure 8:**
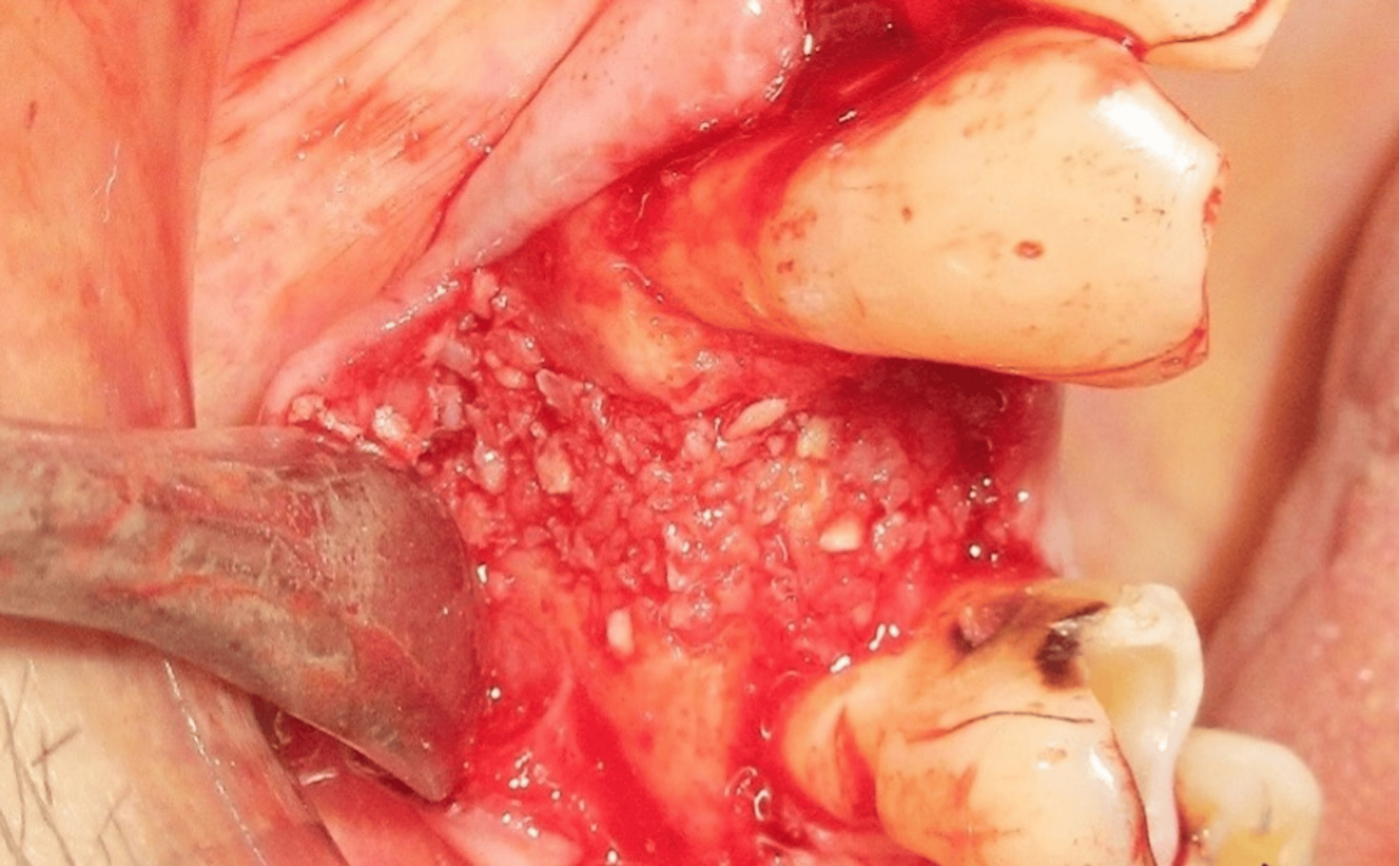
Bioactivated bone graft condensed at the defect site

**Figure 9:**
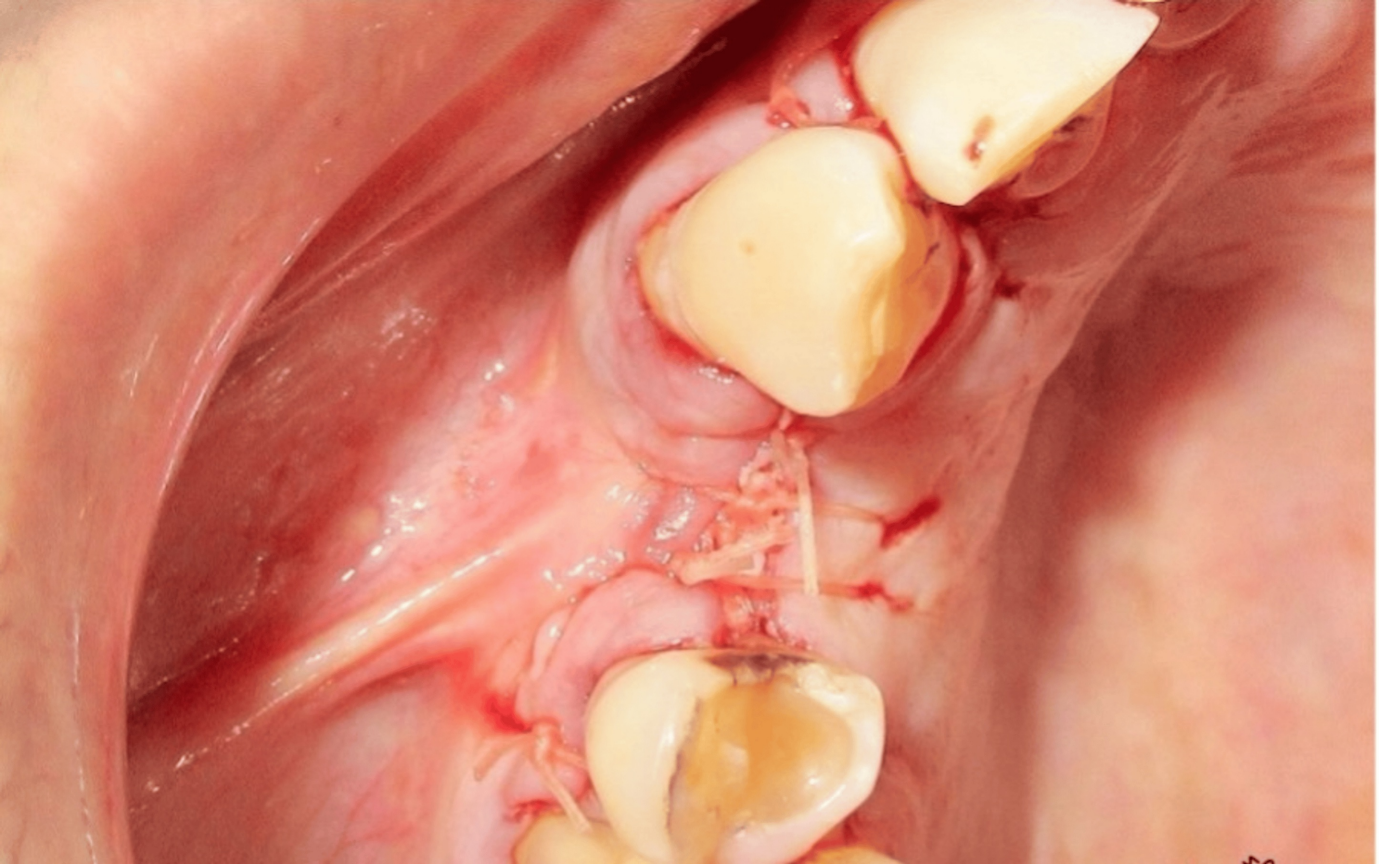
Placement of PGA/PLA sutures PGA, polyglycolic acid; PLA, polylactic acid

**Figure 10:**
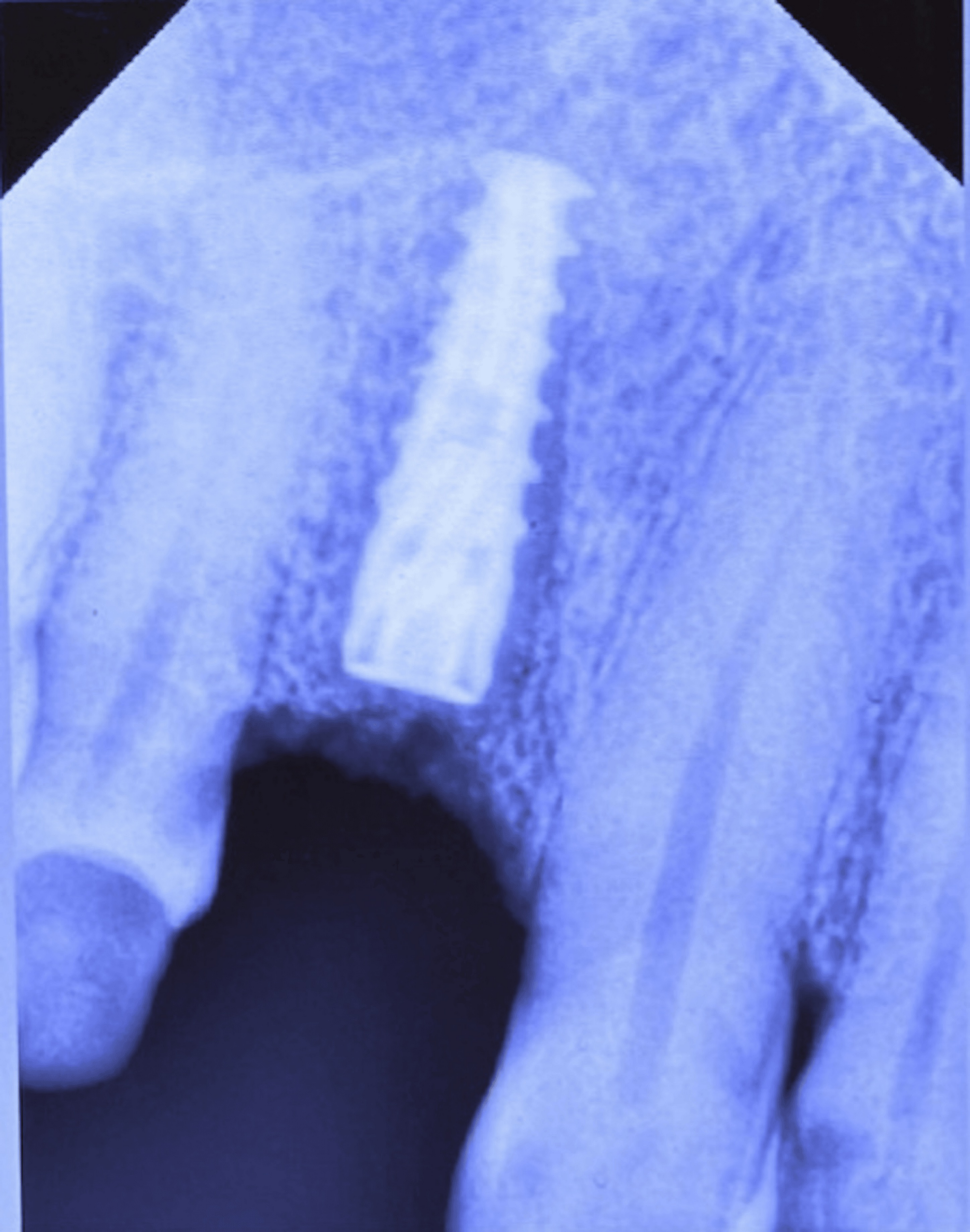
Immediate postoperative radiographic image of the implant

The implant was placed into the osteotomy site, and a cover screw was positioned over it (Figure 6). FDBA, obtained from Tata Memorial Hospital, Mumbai, was mixed with rhBMP-2 (Figure 7). This bioactivated graft was then condensed onto the defect site (Figure 8). The flap was approximated using polyglycolic acid (PGA)/polylactic acid (PLA) sutures (Figure 9). Figure 10 presents the immediate post-operative radiographic image of the implant. 

Postoperative care

The patient was scheduled for follow-up visits at two weeks, four weeks, and five months post-surgery.

At the two-week follow-up, the patient reported minimal pain, and clinical examination showed no signs of infection. The sutures were removed, and the soft tissues exhibited good healing.

At the four-week follow-up, the implant site remained stable. The patient was advised to continue a soft diet for an additional four weeks and to avoid applying excessive force on the implant during this early healing phase.

Second stage surgery

**Figure 11:**
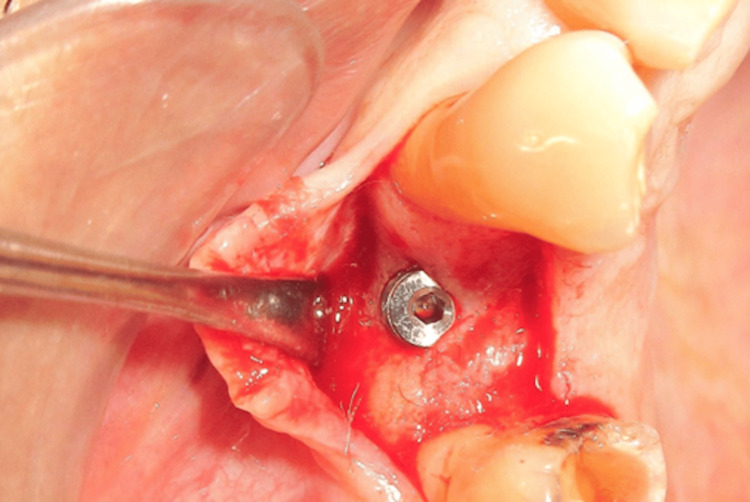
PIBR PIBR, peri-implant bone regeneration

**Figure 12:**
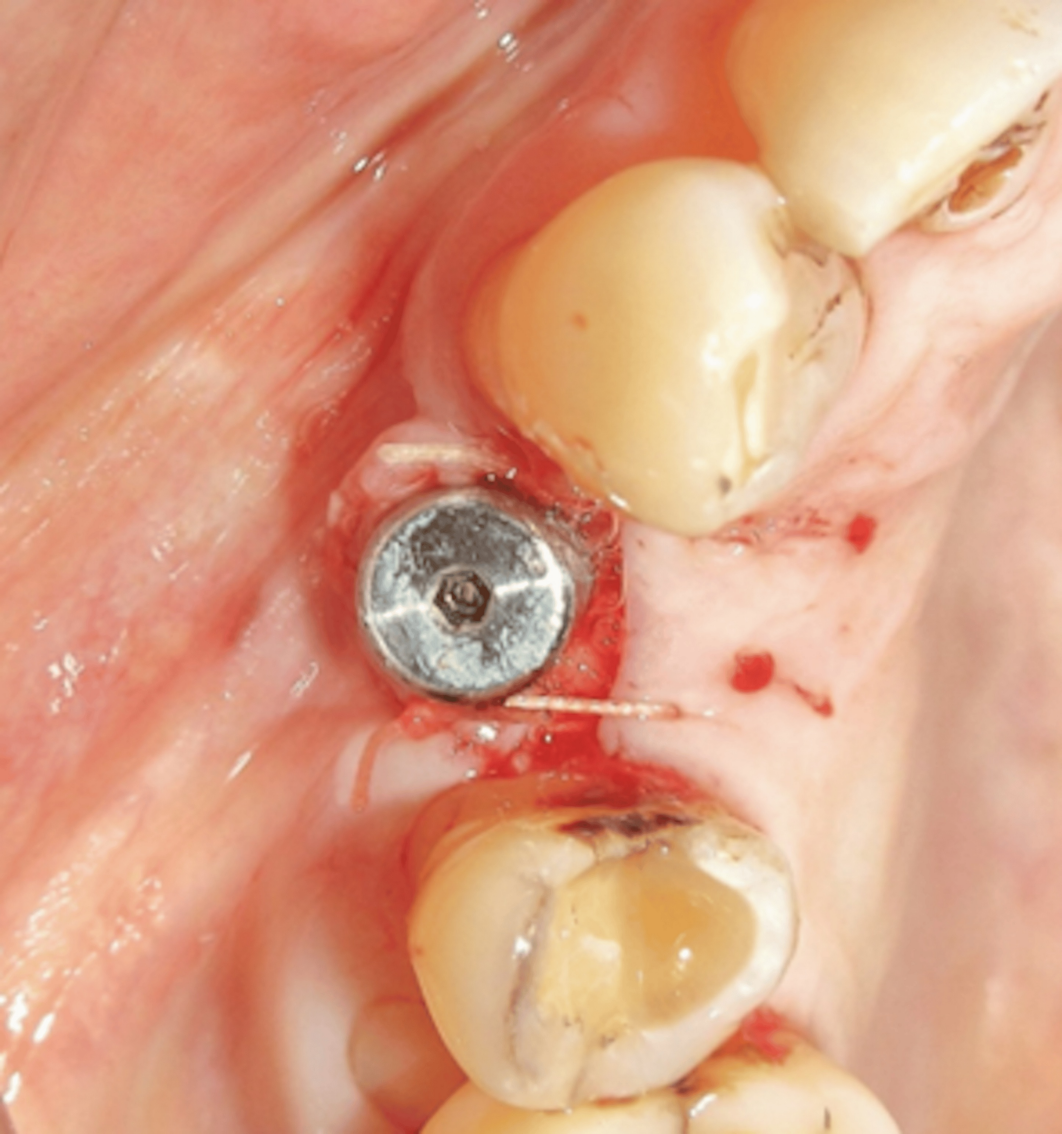
Placement of the gingival former

At the five-month follow-up, a reentry (second-stage) procedure was performed to assess the extent of PIBR. A mid-crestal incision was made under local anesthesia. Clinical examination revealed satisfactory bone fill around the implant, with no signs of peri-implant bone resorption, and the implant remained stable (Figure 11). The cover screw was removed, and a healing abutment (gingival former) was placed. Primary closure was achieved using PGA/PLA sutures (Figure 12).

Final prosthesis

**Figure 13:**
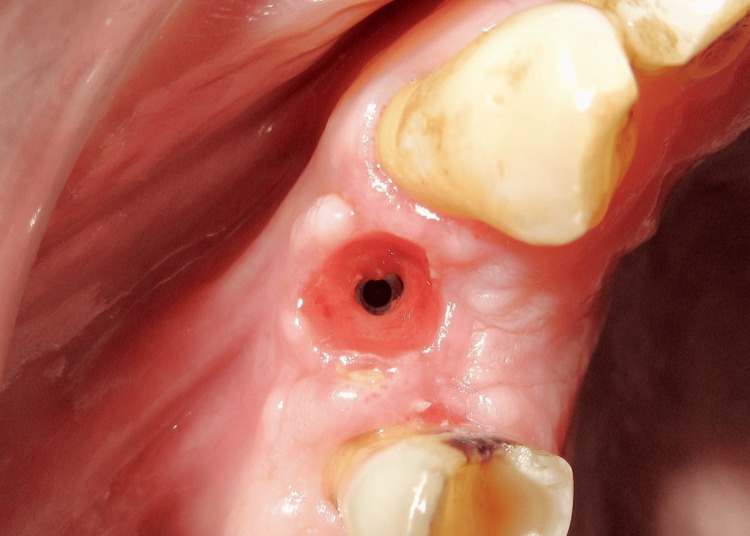
Formation of the peri-implant gingival collar

**Figure 14:**
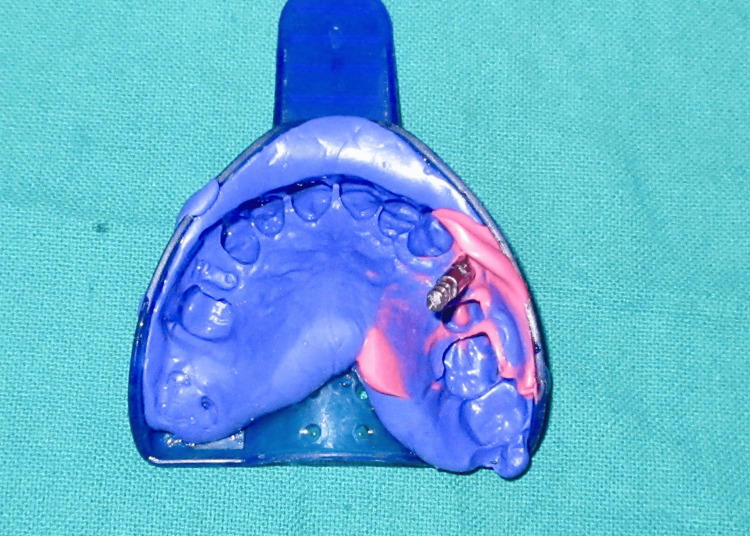
Open tray impression

**Figure 15:**
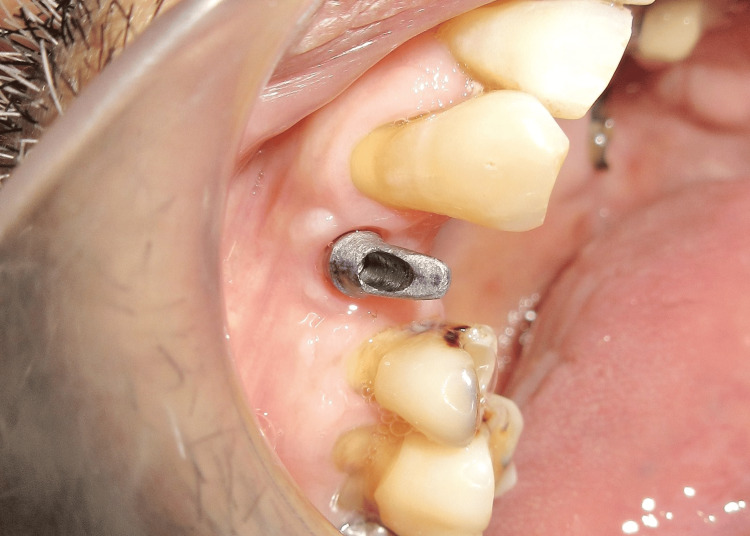
25° abutment with a 3 mm collar height

**Figure 16:**
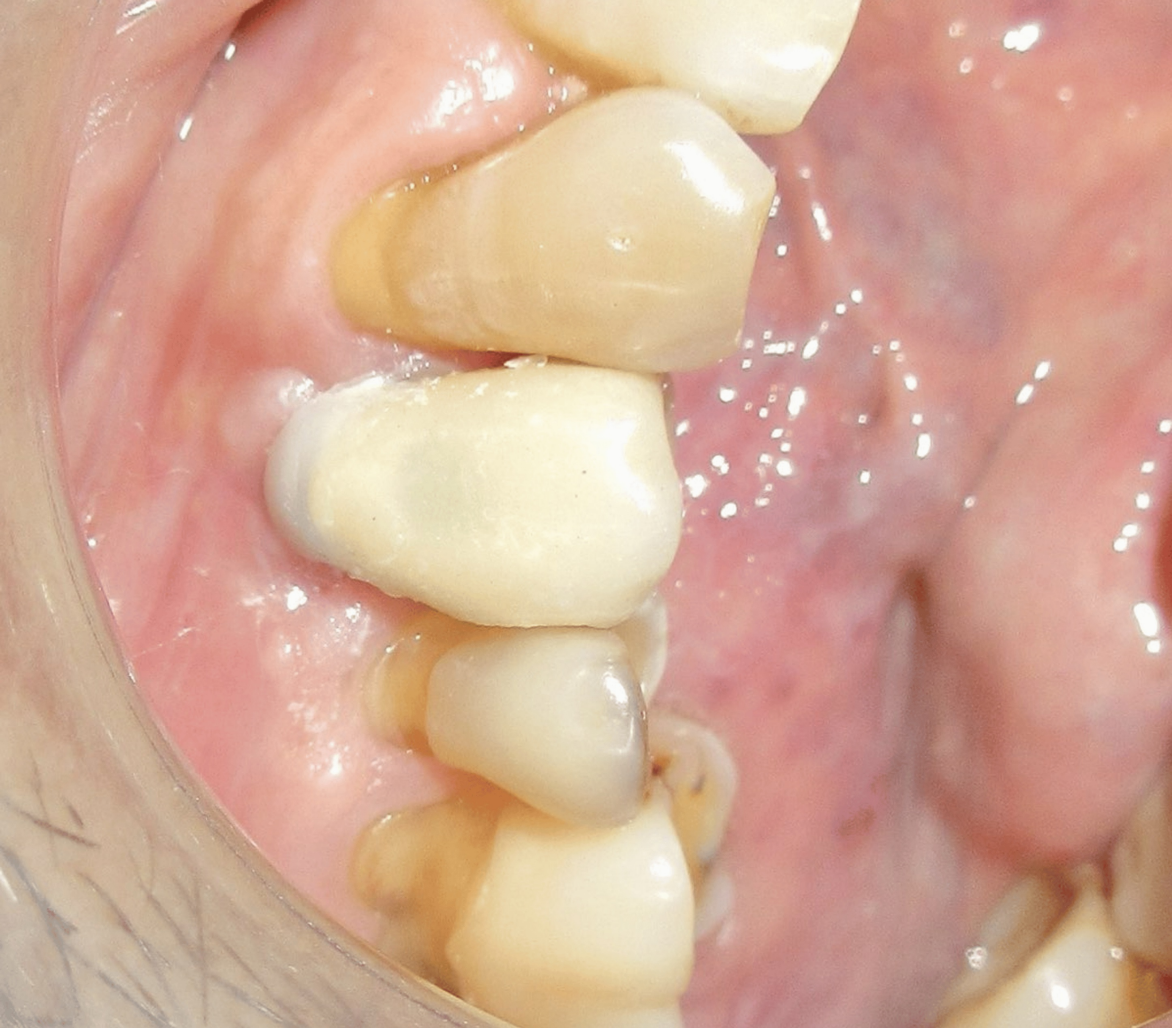
Cement-retained prosthesis delivered successfully

Two weeks after the second stage surgery, the peri-implant gingival collar was evaluated. A well-formed gingival collar with a height of 3 mm was observed (Figure 13). An open tray impression was taken (Figure 14), and a 25° abutment with a 3 mm collar height was selected (Figure 15). The final cement-retained metal-ceramic prosthesis was then delivered (Figure 16). The patient was provided with oral hygiene instructions to ensure the long-term success of the implant.

The patient was satisfied with the functional and aesthetic outcomes. The restoration remained stable, with no signs of implant mobility or discomfort.

Follow-up and results

**Figure 17:**
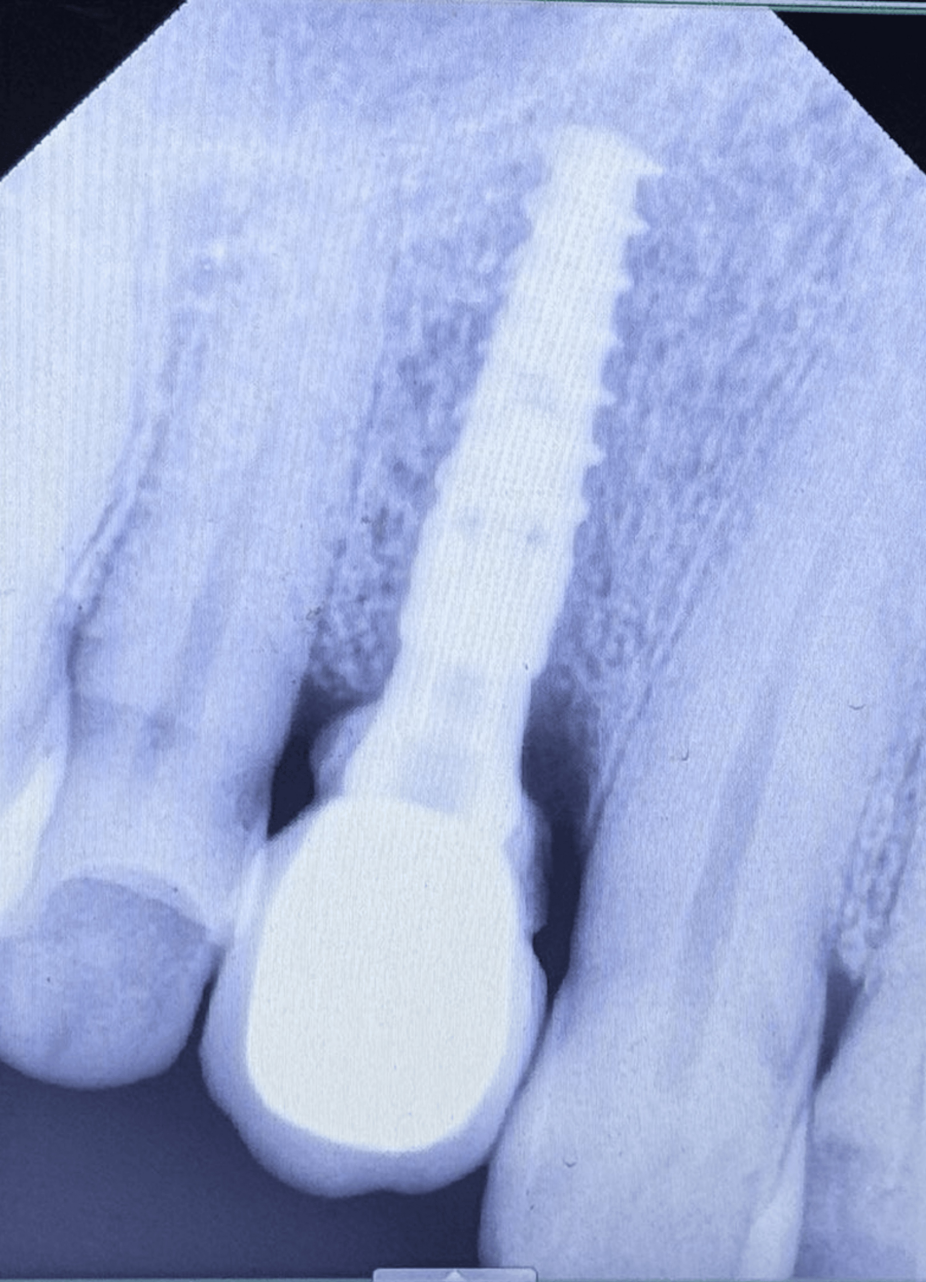
One-year follow-up

At the 12-month follow-up, clinical and radiographic evaluations confirmed continued osseointegration and stable soft tissue around the implant (Figure 17). A thorough examination revealed no clinical or radiographic complications. The prosthetic components remained intact, with no signs of ceramic fracture or abutment loosening. Additionally, no marginal bone loss was observed at the one-year mark.

## Discussion

Immediate implant placement has gained popularity as a treatment modality due to its ability to reduce overall treatment time and preserve bone volume [6]. However, its success depends on several factors, including the quality and quantity of the surrounding bone, the primary stability of the implant, and proper soft tissue management [11].

In cases where bone quality is compromised, additional techniques such as bone grafting, guided bone regeneration, or the application of growth factors may be necessary to enhance healing and ensure long-term implant success [12].

BMPs are naturally occurring growth factors that play a key role in bone formation and healing. Among them, rhBMP-2 has been extensively studied and widely used in both orthopedics and dentistry to promote bone regeneration and stimulate osteogenesis. When applied to dental implants, rhBMP-2 can enhance osseointegration, particularly in sites with insufficient bone volume or compromised healing conditions [13].

In this case, the use of a rhBMP-2-coated implant facilitated bone regeneration around the implant site, leading to successful osseointegration and a stable final restoration. The rhBMP-2 coating likely accelerated the healing process and improved bone-implant contact, reducing the need for additional grafting procedures. Additionally, it provided a biologically active surface that enhanced the implant’s primary stability - an essential factor in immediate implant placement [14].

The findings from this case are consistent with previous studies [8,10] demonstrating the effectiveness of rhBMP-2-coated implants in promoting faster bone healing and osseointegration. Research has shown that rhBMPs significantly improve bone regeneration in cases of implant placement in compromised bone sites, particularly in the posterior maxilla and mandible, where bone volume and density are often limited. Moreover, rhBMP-2-coated implants may shorten healing time compared to conventional implants, allowing for faster restoration of function and aesthetics [10].

In a randomized clinical study, Fiorellini et al. [15] compared two concentrations of rhBMP-2 (1.5 mg/mL and 0.75 mg/mL) delivered with a bioabsorbable collagen sponge carrier to a control group receiving the collagen sponge alone. Their findings confirmed that patients treated with 1.5 mg/mL rhBMP-2 exhibited significantly greater bone formation than those in the other groups.

Similarly, Kao et al. [16] investigated different grafting materials combined with rhBMP-2 for sinus floor augmentation. Their study found that when compared to rhBMP-2 combined with a xenograft, the use of allografts or biphasic calcium phosphate with rhBMP-2 resulted in superior radiographic and histological bone development.

## Conclusions

This case report highlights the successful use of a rhBMP-2-coated implant for immediate implant placement in a patient with a missing maxillary incisor. The application of rhBMP-2 facilitated bone regeneration at the implant site, promoting early osseointegration and reducing the need for additional bone grafting. The patient experienced minimal complications, and the final restoration remained stable with excellent aesthetic outcomes.

Immediate implant placement with rhBMP-2-coated implants presents a promising approach for enhancing dental implant success, particularly in cases with compromised bone quality and volume. However, long-term follow-up is essential. Further research and extended clinical trials are necessary to fully establish the benefits and limitations of this treatment across a broader range of clinical scenarios.
